# Early emergence of motivational and hedonic feeding deficits in the TgF344-AD rat model of Alzheimer’s disease

**DOI:** 10.3389/fnagi.2025.1572956

**Published:** 2025-04-28

**Authors:** Sean B. Ostlund, Grace Chen, Alisa Kosheleff, Lindsay M Lueptow, Irina Zhuravka, Sally A. Frautschy, Hoa A. Lam, Nigel T. Maidment

**Affiliations:** ^1^Department of Anesthesiology and Perioperative Care, Irvine School of Medicine, Irvine Center for Addiction Neuroscience (ICAN), University of California, Irvine, Irvine, CA, United States; ^2^Department of Psychiatry and Biobehavioral Sciences, Semel Institute for Neuroscience and Human Behavior, Hatos Center for Neuropharmacology, University of California, Los Angeles, Los Angeles, CA, United States; ^3^Behavioral Testing Core, Department of Psychology, University of California, Los Angeles, Los Angeles, CA, United States; ^4^Departments of Neurology and Medicine, Geriatric Research Education and Clinical Center, Veterans Greater Los Angeles HealthCare System, University of California, Los Angeles, Los Angeles, CA, United States

**Keywords:** Alzheimer’s, apathy, anhedonia, dementia, avolition

## Abstract

**Introduction:**

Alzheimer’s disease (AD) is characterized by progressive cognitive decline and has a long prodromal phase during which subclinical cognitive deficits and neuropsychiatric symptoms may begin to emerge. Apathy, defined as a lack of motivation or volition, is increasingly recognized as a core feature and a potentially early marker of AD. Despite its significance, apathy-like behavior has been underexplored in transgenic models of AD.

**Methods:**

We performed a longitudinal analysis of apathy-like behavior using the well-established TgF344-AD rat model. We compared male and female TgF344-AD and wildtype rats on hedonic (palatable food intake) and motivational (progressive ratio) assays during early (3—4 months), intermediate (6–7 months), and later (9–10 months) stages of adulthood.

**Results:**

We found that female TgF344-AD rats exhibited early and persistent deficits in motivational and hedonic feeding, emerging at 3–4 months and 6–7 months, respectively. During a battery of cognitive tests conducted after 12–14 months of age, TgF344-AD rats were impaired in spatial working memory but also showed wide-ranging deficits in exploratory behavior, which may also be indicative of an apathy-like loss of investigatory drive.

**Conclusion:**

Our findings highlight the TgF344-AD rat as a valuable model for studying early apathy-like behavior in AD and underscore the need to consider sex differences in AD research to better understand the prodromal phase of this disease.

## Introduction

Alzheimer’s disease (AD) is a leading cause of dementia and morbidity among the elderly (Alzheimer’s Association, 2024), involves progressive and irreversible cognitive dysfunction, and is believed to have a long prodromal phase that begins years or even decades before diagnosis (Morris, 2005; Sperling et al., 2018). By identifying markers of prodromal AD, it may be possible to develop more effective strategies to target and combat this disease early in its progression.

Apathy – commonly defined as a deficit in motivation or self-initiated behavior – is increasingly recognized as a core feature of AD (Lanctôt et al., 2023; Landes et al., 2001). Apathy is the most common neuropsychiatric symptom of AD (Leung et al., 2021; Zhao et al., 2016), and is associated with severity of cognitive impairment as well as functional disability, self-neglect, caregiver distress, and reduced quality of life (Hongisto et al., 2018; Landes et al., 2001; Massimo and Evans, 2014; Mukherjee et al., 2017). Importantly, apathy is a risk factor for rapid and severe cognitive decline (Palmer et al., 2010; Pink et al., 2015; Richard et al., 2012; Robert et al., 2006; Starkstein et al., 2006), and, because it is often observed early in disease progression, is increasingly recognized as a marker of prodromal AD (Delrieu et al., 2015; Johansson et al., 2020). However, despite its high prevalence and clinical significance in AD, there has been relatively little investigation of apathy-like behavior in transgenic models of AD.

Apathy in AD is expressed across multiple dimensions (Lanctôt et al., 2023; Marin, 1991; Robert et al., 2009; Starkstein et al., 2001). In addition to a primary deficit in motivation or volition (Lanctôt et al., 2023), which impacts initiation of purposeful behaviors like meal preparation and self-care, patients with apathy may also display blunted reactions to affective or emotional stimuli, such as a loss of pleasure in normally rewarding activities. These affective and motivational dimensions of apathy can be reliably measured in rodents using established assays of hedonic feeding behavior (Davis and Smith, 1992; Higgs and Cooper, 1998) and willingness to work for food rewards (Le Heron et al., 2019), respectively.

Our study used the well-characterized TgF344-AD rat model of AD (Cohen et al., 2013), which expresses two human genes implicated in familial early-onset AD driven by the mouse prion promoter: the “Swedish” mutant amyloid precursor protein (APPsw) and the presenilin-1 exon 9 deletion (PS1ΔE9). The TgF344-AD line has been extensively studied and displays a full range of AD pathology including β-amyloid plaques, neurofibrillary tangles, gliosis, neuroinflammation, and apoptosis, along with cognitive impairment (Cohen et al., 2013). While these pathological features peak in old age, they show a long and steady age-dependent progression. Evidence of soluble amyloid-β peptide accumulation, tau reactivity, and gliosis appear as early as 6 months of age (Cohen et al., 2013). Although some reports indicate that memory impairment in this model is progressive and emerges at 10 months or later (Berkowitz et al., 2018; Cohen et al., 2013), recent findings suggest that TgF344-AD rats display much earlier cognitive deficits and emotional changes (Bernaud et al., 2022; Hernandez et al., 2022; Hernandez C. et al., 2024; Pentkowski et al., 2022; Saré et al., 2020).

There is also growing evidence that TgF344-AD rats and other transgenic AD models exhibit sex-dependent cognitive impairment, typically with females showing more pronounced cognitive deficits and increased neuropathology (Carroll et al., 2010; Chaudry et al., 2022; Gallagher et al., 2013; Hemonnot et al., 2022; Yang et al., 2018). Such findings may relate to clinical reports that AD is more prevalent, and tends to be more severe, in women (Colombo et al., 2018; Ferretti et al., 2018; Irvine et al., 2012; Sinforiani et al., 2010). Although much remains unknown regarding the influence of sex on expression of apathy-like behavior in rodent AD models, it was recently shown that 8–9 month old TgF344-AD rats show impaired motivation to work for food rewards and that this effect is more pronounced in females (Hernandez C. et al., 2024). However, it is not clear how early such deficits emerge, whether they are accompanied by altered hedonic reward processing, or whether they predict later cognitive function at middle age.

The current study applied a longitudinal design to investigate apathy-like behavior in TgF344-AD rats during a prodromal window, with rats undergoing separate rounds of testing at early (3–4 months), intermediate (6–7 months), and later (9–10 months) stages of adulthood. During each round of testing, we assessed emotional (hedonic feeding task) and motivational (progressive ratio task) responses to a highly-palatable sweetened condensed milk (SCM) reward. After reaching 12–14 months of age, rats were administered a battery of tests (spontaneous alternation, novel object and novel place recognition) to assess their cognitive function.

## Materials and methods

### Subjects and apparatus

We obtained female hemizygous TgF344-AD rats from the Rat Resource and Research Center (RRRC, Columbia Missouri), which were bred in-house with male wildtype F344 rats (Envigo) to generate hemizygous TgF344-AD (Tg) and wildtype (WT) offspring. Genotyping was conducted by PCR using primers and procedures detailed in the RRRC document - RRRC 699. We used 22 Tg (10/12 M/F) and 17 WT (7/10 M/F) rats as experimental subjects, which were run in two separate cohorts (cohort 1 ns: male/Tg = 5; female/Tg = 8; male/WT = 3, female/WT = 3; cohort 2 ns: male/Tg = 6; female/Tg = 3; male/WT = 4, female/WT = 8). Experimental rats were weaned at postnatal day (PND) 21 and group-housed in transparent plastic cages (2–5 per cage) with corncob bedding in a temperature- and humidity-controlled vivarium. Experimental procedures were conducted during the light phase (between 9 a.m. and 5 p.m.) of a 12/12 h light/dark cycle. Unless stated otherwise, rats were provided unrestricted access to home chow and tap water in their home cages. All procedures were conducted in compliance with the National Research Council’s Guide for the Care and Use of Laboratory Animals and were authorized by the UCLA Institutional Animal Care and Use Committee.

Appetitive behavioral procedures took place in eight identical Med Associates operant chambers (East Fairfield, VT) housed in sound- and light-attenuated enclosures. Each chamber had a stainless-steel grid floor and was equipped with a retractable lever located to the left of a recessed food port on the front wall. The lever was extended into the chamber for all instrumental conditioning sessions but was retracted at all other times. When used to reinforce instrumental behavior, 100 μl volumes of sweetened condensed milk (SCM) solution were injected via syringe pump into a cup at the base of the food port. A photobeam detector was used to record food-port entries. In separate sessions, a drinking bottle was placed on the outside of the rear chamber wall, providing rats with unrestricted access to SCM solution via a gravity fed, stainless-steel spout which was accessible through a small hole in the rear wall. A contact lickometer (ENV-250B; Med Associates) was attached to the drinking spout to record individual licking events. A houselight (24 V, 2 W), positioned at the center-top of the rear wall, provided illumination during all experimental sessions. MED-PC IV software was used to control experimental events and record data with 10 ms resolution.

#### Initial unrestricted SCM consumption testing

Once reaching early adulthood (range: PND 68–120), rats were handled once-daily for 5 days before beginning behavioral testing. During the last 3 days of handling, rats were given 4 h of unrestricted access to a bottle of 50% SCM solution (v/v in water) in their home cage to familiarize them with consuming this reward stimulus via a stainless-steel drinking spout. During each of the next 2 days, rats were placed into individual behavioral test chambers for 30-min sessions of unrestricted access to 50% SCM solution (accessible from the same stainless-steel spout through an aperture on the back wall of the test chamber) followed by a series of 30-min consumption test sessions with varying concentrations of SCM solution (2.5%, 10%, 25%, and 50%) in pseudorandom order (Latin square). The stainless-steel spout was then removed from the rear wall of the test chamber until later rounds of unrestricted consumption tests.

#### Progressive ratio testing with SCM

Rats were then put on a brief, mild food restriction schedule (∼10g or ∼12 g of home chow/rat/day for females and males, respectively) and given 2 days of magazine training to familiarize them with consuming SCM rewards from a food cup situated within a recessed food port on the front wall of the test chamber. Each session consisted of 15 × 100 μl volumes of 50% SCM delivered on a 90 s variable-time schedule. Rats were then given instrumental training to lever press for 50% SCM (100 μl) on a fixed-ratio (FR) schedule of reinforcement, with an initial 3 days on an FR-1 schedule (one press required per rewards) and an additional 3 days on an FR-3 schedule (three presses per rewards). These sessions lasted for 30 min or until 30 rewards were earned. The food restriction regimen was then suspended (ad lib home chow was restored) for the remainder of the study beginning immediately after the last FR-3 session. Rats were then given 3 days of progressive ratio (PR) testing in which 50% SCM could be earned on a schedule that progressed arithmetically in 1-press increments with three repetitions per step (beginning after the first increment), such that the response requirement for consecutive rewards was 1, 2, 2, 2, 3, 3, 3, 4, etc.

#### Longitudinal SCM testing

Initial SCM consumption and progressive ratio testing occurred when rats were approximately 3–4 months old (ending PND 101–153), after which they remained undisturbed in their home cages until they were given additional rounds of testing at approximately 6–7 months (ending PND 163–215) and 9–10 months (ending PND 237–289) of age. Rats continued to have unrestricted access to lab chow in their home cages throughout retesting. Rounds 1 and 2 were separated by an average of 67.5 days (range: 57–78), and rounds 2 and 3 were separated by an average of 81 days (range: 76–86). Each new round of testing began with a single 30-min session to refamiliarize them with free-consumption of 50% SCM from a stainless-steel spout at the rear chamber wall, which was followed by four daily free-consumption sessions with varying SCM concentrations, as during initial testing. Rats then received three sessions of PR testing (1 session/day) with 50% SCM delivered into the recessed food port in the front wall, as before. Statistical analyses were performed on data from the final (3*^rd^*) session of PR testing each round.

We also analyzed rats’ average body weight (g) during each of these three rounds of SCM testing.

#### Cognitive testing

Rats remained undisturbed in their home cages until reaching approximately 12–14 months of age (PND 379–424), when they were transferred to the UCLA Behavioral Testing Core for cognitive testing (approximately 130 days (range 124–136) after the last PR test). They were given 1 week to acclimate and were handled 5–7 days prior to further behavioral testing, which was conducted under low ambient white light conditions (∼20 lux) with additional red light to enhance video recording. Run order was fully randomized across sex and genotype conditions. All cognitive testing equipment and stimuli were cleaned and sanitized (70% ethanol) between animals and before and after each day of testing (*Strike Bac* germicidal cleaner). Video recordings were made of all test sessions and analyzed as described below by blinded experimenters.

Rats were first administered a *spontaneous alternation test*. Briefly, each rat was placed at the distal end of one of three arms (30 cm length from center door, 10 cm width, 20 cm height) of the Y-maze (Pathfinder Maze System; Lafayette Instrument Co; transparent plexiglass walls and black flooring) and allowed to voluntarily enter the center chamber (33 cm diameter; doors 12 × 10 cm), where they were then confined for a 30-s waiting period. At this point, all doors were opened and the rat was allowed to freely explore all three arms for 8 min, before being returned to their home cage. The total number of entries (both hind paws cross the entrance to arm) and spontaneous alternation% [total alternations/(total entries–2) × 100] were quantified. An alternation is defined as the successive entry into each of the three, without a repeat visit (so ABC, CBA, ACB, but not ABA, ACA, BCB, etc.) Spontaneous alternation% was arcsine transformed for statistical analysis. For these analyses, we excluded three rats (two male Tg and one male WT) that made 0 total alternations to reduce variability. These rats were included in subsequent analyses.

After a rest period of 5–7 days, rats were given 2 days of habituation (10 min) to an open field chamber (80 cm length × 40 cm width × 40 cm height; gray walls and black flooring). Each session began by placing the rat in the center of the chamber (facing a random direction). Locomotor activity (meters traveled) during these sessions was quantified using ANY-maze software (v. 5; Stoelting). On the following day, rats were tested for *object in place* and *object recognition* memory. This began with a 10-min training session, during which rats were placed in the center of the chamber (random direction) and allowed to freely explore two rat-sized objects (e.g., plastic soap dispenser, sippy cup, 500 ml Erlenmeyer flask, etc.) placed in adjacent quadrants on one end of the open field (10 cm from corner). Object identity and place assignments were randomized and counterbalanced with sex and genotype. No consistent baseline preferences were apparent. After a 15-min waiting period in the home cage, rats were returned to the center of the open field for 10 min to explore the same two objects, with one object remaining in the training quadrant and the other object moved to a novel quadrant (diagonal to the alternate object). The amount of time exploring each object (defined as orienting with nose within 3 cm of the object) during initial training and object-in-place testing was hand-scored by a blinded experimenter using a stopwatch. These data were used to compute a discrimination index of the relative time spent exploring the moved versus unmoved object [(moved–unmoved)/(moved + unmoved)]. This analysis focused on the first 5 min of the test, when investigatory behavior was greatest. After waiting an additional 15 min in the home cage, rats were returned to the center of the open field for a final 10-min test of object recognition. Briefly, the familiar object that had been moved remained located in the new quadrant and the other (previously unmoved) object was replaced with a novel object. Time spent exploring each object was again hand-scored and used to compute a discrimination index of relative time spent exploring the novel versus familiar object [[(novel–familiar)/(novel + familiar)] (first 5 min). Data from rats that spent less than 20 s actively exploring either object during training or testing were excluded from object-in-place (one male Tg and one male WT) and novel object recognition testing (one male Tg, same animal as for object-in-place). These rats differed from those excluded from spontaneous alternation testing.

### Statistical analysis

Data were analyzed with JASP (0.18.3). Repeated ANOVAs were used to analyze hedonic feeding (*between-subjects factors*: genotype and sex; *within-subject factors*: time-bin/concentration and age), PR performance (*between-subjects factors*: genotype and sex; *within-subject factor*: age) and body weight (*between-subjects factors*: genotype and sex; *within-subject factor*: age). Two-way ANOVAs (genotype and sex) were used to analyze data from individual cognitive tests. Significance was set at *p* < 0.05 for all tests. Data from cohorts 1 and 2 were combined after preliminary analyses found no significant cohort × genotype or cohort × genotype × sex interactions. Pearson’s correlations were assessed to investigate associations between PR performance at 3–4 months and cognitive test performance at 12–14 months. Data are presented as means ± standard error of the means (SEMs). Final group Ns are provided for each analysis in the figure captions.

## Results

We conducted a longitudinal analysis of hedonic and motivational responses to SCM rewards in male and female TgF344-AD rats at three ages spanning early to middle adulthood. Consistent with earlier reports (Bernaud et al., 2022; Hernandez et al., 2022; Rutkowsky et al., 2024; Srivastava et al., 2023), Figure 1A shows that TgF344-AD rats weighed more than wildtype controls (F_1,36_ = 12.16, *p* < 0.001), an effect that did not interact with sex or age (Fs < 1), though males weighed more than females (F_1,36_ = 739.71, *p* < 0.001) and gained weight faster over time (age: F_2,72_ = 429.17, *p* < 0.001; age x sex interaction: F_2,72_ = 95.24, *p* < 0.001; no other significant effects or interactions).

**FIGURE 1:**
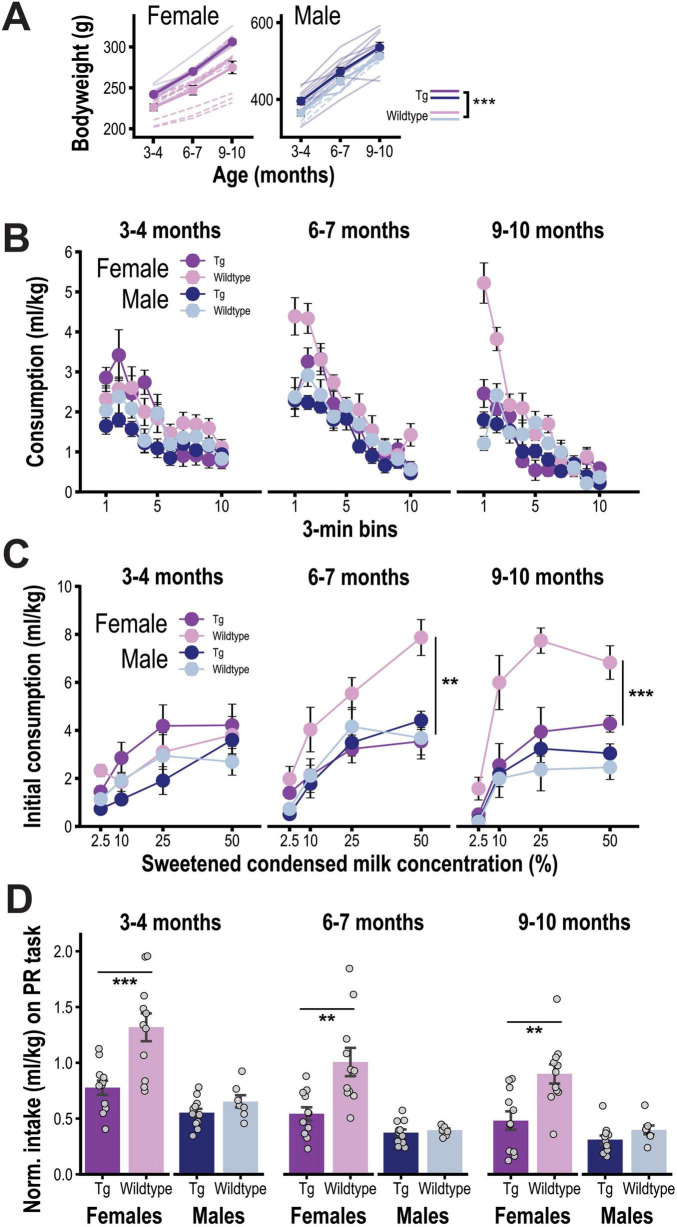
**(A)** Mean free-feeding bodyweight (± SEM) for female (left) and male (right) TgF344-AD (Tg) and wildtype (WT) rats. Data are plotted across ages 3–4, 6–7, and 9–10 months as in subsequent panels. **(B)** Mean bodyweight-normalized sweet condensed milk (SCM) consumption (ml/kg; ± SEM) over successive 3 min bins during free-access tests conducted longitudinally across ages for male and female Tg and WT rats. Consumption rates have been collapsed across SCM concentration (four levels/age) to highlight the within-session pattern of intake over time. **(C)** Mean bodyweight-normalized SCM consumption (ml/kg; ± SEM) during the first 3 min of active SCM consumption across SCM concentration and across ages for male and female Tg and WT rats. **(D)** Mean bodyweight-normalized intake (ml/kg; ± SEM) of response-contingent SCM rewards during progressive ratio tests conducted across ages for male and female Tg and WT rats. Group sizes for all analyses were as follows: Female Tg (*n* = 11), Female WT (*n* = 11), Male Tg (*n* = 11), and Male WT (*n* = 7). ** *p* < 0.01, *** *p* < 0.001.

Figure 1B presents the temporal pattern of bodyweight-normalized SCM intake (ml/kg) within free-feeding consumption tests, collapsed across SCM concentrations, which shows that the rate of intake peaked early in the session before entering a steady decline phase as satiety developed. These data also reveal a sex- and age-dependent deficit in initial SCM intake that emerged as female Tg rats aged (age × genotype × sex × time bin: F_18_,_648_ = 3.14, *p* < 0.001). Importantly, the early phase of intake is predominantly driven by orosensory rewards mechanisms and therefore provides an assay of the emotional-hedonic response to taste stimuli (Davis, 1989). As in prior studies (Halbout et al., 2023; Halbout et al., 2024; Marshall et al., 2017), we isolated hedonic feeding behavior by computing SCM intake during the first 3 min of active feeding within each session (i.e., after first contact with SCM but before satiety induction). Consistent with this interpretation, Figure 1C shows that early intake increased with SCM solution concentration, or palatability (F_3,108_ = 61.35, *p* < 0.001) across all age periods (3–4 months: F_3,108_ = 8.51, *p* < 0.001, 6–7 months: F_3,108_ = 39.21, *p* < 0.001, 9–10 months: F_3,108_ = 32.96, *p* < 0.001). Early intake was higher in females (sex: F_1,36_ = 43.14, *p* < 0.001), particularly as rats aged (sex × age interaction: F_2,72_ = 6.00, *p* = 0.004), consistent with prior research (Feigin et al., 1987; Hankosky et al., 2018; Marshall et al., 2017; Tapia et al., 2019; Valenstein et al., 1967). More importantly, there was evidence of sex-dependent deficit in Tg group (genotype × sex interaction: F_1,36_ = 13.16; *p* < 0.001) that emerged over time (age × genotype × sex interaction: F_2,72_ = 10.79, *p* < 0.001) and varied with SCM concentration (age × genotype × sex × concentration: F_6,216_ = 3.01, *p* = 0.008). No deficit was observed at 3–4 months (genotype: F < 1; genotype × sex F_1,36_ = 1.83, *p* = 0.19) but was expressed in a sex-dependent manner at 6–7 months (genotype x sex: F_1,36_ = 9.94, *p* < 0.01; genotype x sex x concentration: F_3,108_ = 4.11, *p* < 0.01) and 9–10 months (genotype × sex: F_1,36_ = 20.69, *p* < 0.001; genotype × sex × concentration: F_3,108_ = 2.58, *p* = 0.057). Specifically, female Tg rats showed a progressive attenuation in SCM intake, relative to wildtypes, that was not apparent at 3–4 months (F_1,20_ = 1.14; *p* = 0.30) but was significant at 6–7 months (genotype: F_1,20_ = 20.30, *p* < 0.001; genotype x concentration: F_3,60_ = 4.34, *p* < 0.01) and 9–10 months (genotype: F_1,20_ = 20.35, *p* < 0.001; genotype x concentration: F_3,60_ = 2.13, *p* = 0.11). Male Tg rats did not significantly differ from wildtypes at 3–4 or 6–7 months (ps > 0.33) but consumed SCM at a marginally lower rate at 9–10 months (genotype: F_1,16_ = 4.12, *p* = 0.06).

Each assessment of hedonic feeding was followed by a round of instrumental PR testing to probe rats’ willingness to exert effort for SCM rewards. Once again, the data were normalized for variation in bodyweight (SCM earned on the PR task; ml/kg), though analyses performed on total presses or rewards earned produced similar results (data not shown). As shown in Figure 1D, we found evidence of a sex-dependent apathy-like effect in Tg rats (genotype × sex: F_1,36_ = 8.56, *p* = 0.006; genotype: F_1,36_ = 15.42, *p* < 0.001; sex: F_1,36_ = 31.81, *p* < 0.001), which was apparent across all age periods even though response levels dropped for all groups over time (age: F_2,72_ = 40.04, *p* < 0.001; interactions with age ps > 0.20). During the first round of testing, at 3–4 months, we observed a significant genotype effect (F_1,36_ = 13.53, *p* < 0.001) that interacted with sex (F_1,36_ = 6.43, *p* = 0.02), with female (*p* < 0.001) but not male (F_1,16_ = 2.16, *p* = 0.16) Tg rats earning less SCM through PR performance than sex- and age-matched wildtype controls. Similar effects were detected at 6–7 months (sex × genotype: F_1,36_ = 7.64, *p* = 0.01; genotype for females: F_1,20_ = 11.32, *p* < 0.01; genotype for males: F < 1) and 9–10 months (sex × genotype: F_1,36_ = 4.95, *p* = 0.03; genotype for females: F_1,20_ = 11.42, *p* < 0.01; genotype for males: F_1,16_ = 2.15, *p* = 0.16).

**FIGURE 2:**
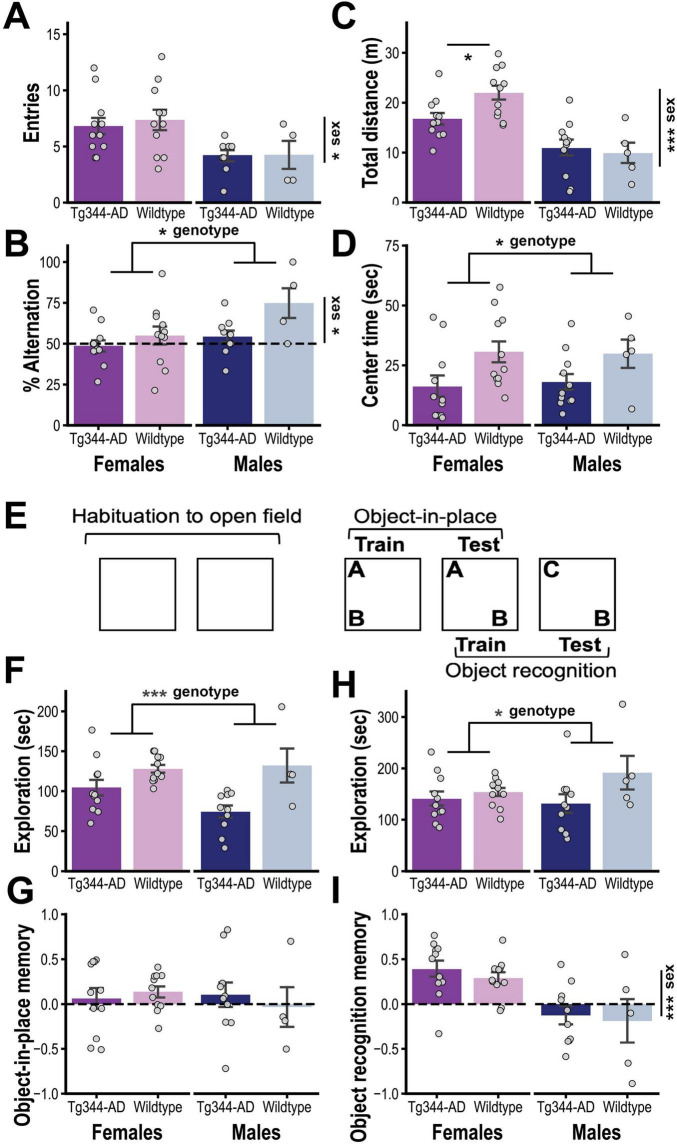
**(A)** Mean number of arm entries (± SEM; top) and **(B)** mean percentage of alternations (± SEM; bottom) in the Y-maze test for TgF344-AD [Tg: female (*n* = 11) and male (*n* = 9)] and Wildtype rats [WT: female (*n* = 11) and male (*n* = 4)]. **(C)** Mean (± SEM) distance traveled and **(D)** time in center during the first session of open field habituation for Tg [female (*n* = 11) and male (*n* = 11)] and WT rats [female (*n* = 11) and male (*n* = 5)]. **(E)** Schematic representation of design for object-in-place and object recognition testing (see text for procedural details). **(F)** Mean (± SEM) time spent exploring the two novel objects during the object-in-place training session and **(G)** the relative time spent exploring the object moved to a novel location at test [(discrimination index = (moved–unmoved)/(moved + unmoved)] **(G)** for Tg [female (*n* = 11) and male (*n* = 10)] and WT rats [female (*n* = 11) and male (*n* = 4)]. **(H)** Mean (± SEM) time spent exploring the two objects during object recognition training and **(I)** the percentage of total time spent exploring the novel object at test [(discrimination index = (novel–familiar)/(novel + familiar)] for Tg [female (*n* = 11) and male (*n* = 10)] and WT rats [female (*n* = 11) and male (*n* = 5)]. * *p* < 0.05, ** *p* < 0.01, *** *p* < 0.001.

After reaching 12–14 months of age, the rats underwent a final round of behavioral testing to assess cognitive function. We first performed a test of spontaneous alternation, a measure of spatial working memory. Figures 2A, B show that Tg rats did not significantly differ from controls in total number of arm entries they made at test (genotype: F_1,31_ = 1.14, *p* = 0.30; sex: F_1,31_ = 34.13, *p* < 0.001; genotype x sex: F_1,31_ = 0.68, *p* = 0.42), but displayed a significant reduction in their tendency to spontaneously alternate between arms (F_1,31_ = 7.42; *p* = 0.01; sex: F_1,31_ = 6.01, *p* = 0.02; sex x genotype: F_1,31_ = 2.77, *p* = 0.11), indicating a deficit in spatial working memory.

Rats were then habituated to the open field that would be used in subsequent tests (see Figure 2E). As seen in Figure 2C, during the first habituation session, there was a non-significant trend indicating a sex-specific reduction in exploration in the Tg group (total distance traveled; genotype x sex: F_1,34_ = 3.35, *p* = 0.076; sex: F_1,34_ = 27.80, *p* < 0.001; genotype: F_1,34_ = 1.52, *p* = 0.23). *Post hoc* analysis (Holm corrected) indicated that female (*p* < 0.05) but not male (*p* > 0.7) Tg rats traveled less total distance than their respective controls. Moreover, regardless of sex, Tg rats spent less time in the center of the open field (genotype: F_1,34_ = 7.27, *p* = 0.01; sex and genotype x sex: Fs < 1), as shown in Figure 2D.

Rats were then trained on an object-in-place task, which began by allowing them to investigate two novel objects (A and B; see Figure 2E). Tg rats spent less time investigating these objects (genotype: F_1,32_ = 14.03, *p* < 0.001; sex: F_1,32_ = 1.45, *p* = 0.24; genotype x sex: F_1,32_ = 0.12; Figure 2F) but were not significantly impaired in selectively investigating the displaced object (Object B) during a subsequent object-in-place memory test (genotype and sex effects and genotype x sex interaction: Fs < 1; Figure 2G). This test session also served as training for object recognition memory. Although the Tg group spent less time exploring the two now familiar objects during this session (genotype: F_1,33_ = 4.34, *p* = 0.045; sex: F_1,33_ = 0.66, *p* = 0.42; sex x genotype: F_1,33_ = 1.80, *p* = 0.19; Figure 2H), they were unimpaired in exploring the novel object during the object recognition memory test (genotype and genotype x sex: Fs < 1; Figure 2I). However, it should be noted that males displayed generally poor performance during the latter test (sex: F_1,33_ = 17.36, *p* < 0.001), which, together with the relatively low final size in the wildtype male subgroup (*n* = 5), makes it difficult to draw conclusions about the effect of genotype in this sex.

**FIGURE 3:**
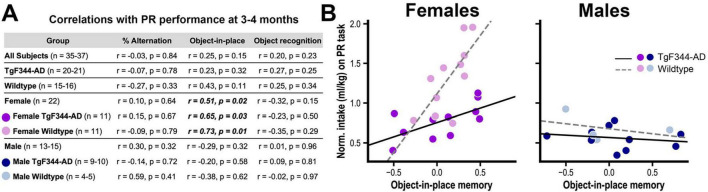
**(A)** Summary of results from correlational analysis between progressive ratio (PR) performance at 3–4 months and cognitive test performance at 12–14 months. Significant relationships are in bold italic font. **(B)** Scatterplots and regression lines for each subgroup showing significant positive correlations between progressive ratio (PR) performance and object-in-place memory for female TgF344-AD and wildtype rats but not for males of either genotype.

We then assessed whether motivational deficits displayed during early adulthood predicted cognitive performance at middle age (Figure 3). PR performance (mg/kg) at 3–4 months was not correlated (Pearson’s, 2-tailed) with spontaneous alternation or novel object recognition scores at 12–14 months in any condition (Figure 3A). However, PR performance was positively correlated with object-in-place recognition memory in female rats, an overall association that was also observed in both Tg and WT female subgroups but not in either male subgroup (Figure 3B). Thus, for female rats, reduced motivation early in adulthood tended to predict poor memory for object locations later in life.

## Discussion

This longitudinal study examined the influence of sex and age on instrumental progressive ratio performance (motivation) and hedonic feeding behavior (emotion) in the TgF344-AD rat model. We observed motivational deficits as early as 3–4 months and hedonic deficits by 6–7 months, effects that were largely restricted to females. TgF344-AD rats also exhibited a cognitive deficit in spatial working memory as well as more widespread reductions in investigatory behavior when tested at 12–14 months.

Pathology associated with AD can begin many years before a clinical diagnosis is made (Braak et al., 2011; Vickers et al., 2016). During this lengthy prodromal phase, there may be evidence of more modest cognitive and executive deficits (Morris, 2005; Sperling et al., 2018) as well as a range of other neuropsychiatric symptoms, with apathy being the most common (Sherman et al., 2018). The core feature of apathy is diminished motivation, with symptoms impacting different domains, including loss of self-initiated goal-directed behavior or cognitive activity and blunted emotional reactivity (Robert et al., 2009). Apathy in AD patients is associated with reduced quality of life (Yeager and Hyer, 2008) and higher caregiver stress (Landes et al., 2001; Mukherjee et al., 2017), and apathy in the prodromal phase is associated with rapid disease progression (Feldman et al., 2007) and deficits in daily functioning (Tam et al., 2008).

Our findings add to a growing body of evidence that TgF344 rats and other rodent AD models display signs of apathy, including deficits in nest-building (Keszycki et al., 2023; Robinson et al., 2024), social interaction (Kosel et al., 2020), and spontaneous locomotor activity (Pardossi-Piquard et al., 2016). This latter finding mirrors clinical data linking low motor activity with apathy in Alzheimer’s patients (David et al., 2012), and is generally consistent with the reduced exploratory behavior displayed by TgF344-AD rats in the current study and similar findings in the literature (Galeano et al., 2014; Saré et al., 2020). There is also evidence that deficits in locomotor activity are more severe for female TgF344 AD rats (Saré et al., 2020) and AD mice (Bourgeois et al., 2018), which is also in keeping with our finding that female TgF344-AD rats showed less activity when first exploring an open field. While such findings may reflect an apathy-related deficit in exploratory drive (Batrancourt et al., 2019; Jackson et al., 2021), prior reports indicate that TgF344-AD rats exhibit heightened fear and anxiety behaviors (Hernandez et al., 2022; Pentkowski et al., 2022; Wu et al., 2020), which may interfere with their investigative activity. Indeed, as in other recent studies (Reitz et al., 2024; Wu et al., 2020), we found that TgF344-AD rats spent less time exploring the center of the open field, potentially reflecting an increase in anxiety-induced thigmotaxis.

Prior studies using the TgF344-AD rat model have also observed deficits in palatable food consumption and food-reinforced behaviors (Hernandez C. et al., 2024; Muñoz-Moreno et al., 2018; Tournier et al., 2021). For instance, Hernandez C. et al. (2024) compared these rats with wildtype controls on a progressive ratio task at approximately 8.5 months of age and found evidence of a sex-dependent motivational deficit that was greater in females. The current study bolsters this finding and demonstrates that this deficit arises as early as 3–4 months and persists longitudinally through at least middle adulthood (9–10 months). Our findings are also consistent with earlier work with 3xTg-AD mice showing that females exhibit more pronounced deficits in food-reinforced tasks (Fertan et al., 2019; Gür et al., 2019).

We assessed feeding and food motivation in the absence of food restriction to focus on hedonic reward processing and minimize the role of homeostatic (hunger) processes. It is therefore notable that Hernandez C. et al. (2024) found similar results in rats maintained on mild food restriction (85% free feeding body weight), indicating that the motivational impairment displayed by female TgF344-AD rats is robust and not heavily influenced by altered satiety/hunger processing, which helps address potential confounds related to their high body weight relative to age-matched WT females (Bernaud et al., 2022; Hernandez et al., 2022; Rutkowsky et al., 2024; Srivastava et al., 2023). Moreover, unlike in the current study, Hernandez C. et al. (2024) tested rats during the active phase of their daily light-dark cycle, suggesting that this variable is not a major factor regulating observed motivational deficits. Although prior studies with this model have found emotional and cognitive impairments regardless of whether rats were tested during active (Hernandez et al., 2022; Hernandez C. et al., 2024; Pentkowski et al., 2022) or rest phases (Bernaud et al., 2022; Tournier et al., 2021; Wu et al., 2020), systematic investigation of this issue may be warranted, particularly given evidence that TgF344-AD rats exhibit altered sleep/wake cycles (Kelberman et al., 2022; Kreuzer et al., 2020).

Consistent with prior research (Feigin et al., 1987; Hankosky et al., 2018; Marshall et al., 2017; Tapia et al., 2019; Valenstein et al., 1967), we found that WT female rats displayed higher levels of palatable food consumption and food-reinforced progressive ratio performance than age-matched WT males. Such findings are of particular interest given the increased prevalence of binge-eating and other eating disorders in females, which is thought to be at least partly mediated by ovarian hormone activity (Ma et al., 2020). It is therefore notable that deficits in feeding and food seeking were restricted to female TgF344-AD rats and appeared to counter the otherwise elevated levels of these behaviors in age-matched WT females, a pattern that was also apparent in the Hernandez C. et al. (2024) study. Further research will be needed to examine whether these deficits are regulated by progesterone and estrogen, which have been shown to protect against development of AD-like neuropathologies and cognitive impairment in female 3xTg-AD mice (Carroll et al., 2007).

Prior research may shed light on potential neural mechanisms underlying AD-related deficits in reward processing and motivated behavior. Hernandez C. et al. (2024) found that AD-like pathology in the prelimbic cortex and basolateral amygdala of TgF344-AD rats predicted deficits in reward optimization on an intertemporal choice task, although no links to motivation on the progressive ratio task were identified. Other studies suggest that motivational deficits in AD may relate to mesolimbic dopamine system dysfunction (Cordella et al., 2018). For instance, the Tg2576 transgenic AD mouse model shows evidence of ventral tegmental area dopamine neuron death and attenuated nucleus accumbens dopamine release by 6 months of age, which is accompanied by deficits in palatable food rewards processing and consumption (Nobili et al., 2017). Although that study used males exclusively, a similar deficit in mesolimbic dopamine release was observed in the APP/PSEN1 mouse model of AD with no indication of sex dependence (Consoli et al., 2021). Interestingly, TgF344-AD rats also show evidence of depressed dopamine release (Ceyzériat et al., 2021), and there is compelling clinical evidence of dopamine dysfunction in prodromal Alzheimer’s disease (D’Amelio et al., 2018; Sasaki, 2018). Future studies will be needed to determine whether dopamine dysfunction underlies the female-specific deficits in motivation and/or hedonic feeding behavior displayed by TgF344-AD rats in the current study. However, while dopamine plays a critical role in regulating effortful, motivated behavior (Ostlund et al., 2012; Salamone and Correa, 2012), it is not strongly involved in hedonic control over feeding behavior (Cannon and Palmiter, 2003; Marshall et al., 2018; Wassum et al., 2011), which raises the possibility that other, dopamine-independent mechanisms are also involved.

While there are reports to the contrary (Colombo et al., 2018), a recent meta-analysis found that apathy is more prevalent in males than females with AD or related dementias (Eikelboom et al., 2022). However, female AD patients have a higher prevalence of depression and other affective disorders (Eikelboom et al., 2022). This is notable since a major symptom of depression is anhedonia, or the inability to experience pleasure from normally rewarding activities, which can be difficult to distinguish from the affective dimension of apathy (Lanctôt et al., 2023; Starkstein et al., 2001). Indeed, attempts to operationalize apathy have emphasized the negative motivational and behavioral symptoms (i.e., reduced self-initiated, purposive behavior), with affective symptoms playing a secondary role in diagnosis (Robert et al., 2009). Even though the PR task used here involves the expression of a self-initiated, purposive action, it is motivated by a palatable food rewards and may therefore also reflect changes in hedonic reward processing. Our finding that female, but not male TgF344-AD rats, were impaired in both the PR task and the hedonic feeding task suggests a common reward processing deficit may underlie both effects as the most parsimonious account. The loss of intrinsically motivated actions, like exploring novel places or objects, may provide a more selective measure of apathy in rodents. Not only do AD patients with apathy show deficits in novelty processing and novelty-seeking behavior (Bastin et al., 2019; Chau et al., 2015; Daffner et al., 1992), similar deficits have been observed in transgenic rodent models of AD (Zufferey et al., 2013), including the TgF344-AD rats (Saré et al., 2020), supported by our observation of reduced exploratory behavior in the current study.

As noted above, we found that TgF344-AD rats displayed a sex-independent deficit in spontaneous alternation, an assay of spatial working memory, during tests conducted at an average of 13.5 months of age. Interestingly, prior research with this AD model have found deficits on this task at 24 months (Cohen et al., 2013) but not between 7 and 10 months (Reitz et al., 2024; Tournier et al., 2021), although 7 months-old TgF344-AD rats were found to be more vulnerable to impairment caused by adolescent ethanol exposure (Reitz et al., 2024). Such findings are consistent with other evidence of progressive cognitive impairment in this model (Berkowitz et al., 2018; Bernaud et al., 2022; Cohen et al., 2013). However, we did not detect significant deficits in object-in-place or novel object recognition memory. The literature on TgF344-AD rats using these tasks have mixed results, with some (Cohen et al., 2013; Galloway et al., 2018; Morrone et al., 2020) but not other studies (Chaney et al., 2021; Galloway et al., 2018; Goodman et al., 2021; Hernandez A. et al., 2024; Ratner et al., 2021; Wu et al., 2020) finding impairments. However, we did find evidence that reduced motivation for rewards early in adulthood predicted poor performance on the object-in-place task at middle age, an association that was apparent in WT and Tg females but not males of either genotype. Future research will be needed to explore this relationship, particularly given established links between apathy and cognitive dysfunction in AD (Delrieu et al., 2015; Johansson et al., 2020; Palmer et al., 2010; Pink et al., 2015; Richard et al., 2012; Robert et al., 2006; Starkstein et al., 2006). Importantly, we found no evidence that motivation was correlated with either object recognition or spontaneous alternation effects, which is in line with a similar finding by Hernandez C. et al. (2024).

Our longitudinal approach allowed us to track changes in hedonic feeding and motivation over time and assess links to later cognitive function, but this design also has limitations. For instance, we found that WT females increased their palatable food intake over time. The group specificity of this effect suggests that it was driven by sex- and age-dependent changes in hedonic processing, though it is possible that repeated experience with the task and SCM rewards had an influence. In contrast, all groups showed declining levels of PR performance over time. This lack of group-specificity suggests that rats, in general, learned to exert less effort for SCM rewards over repeated cycles of testing. Given this potential for learning-related behavioral change, we focused our analysis on whether the performance of TgF344-AD rats differed from age- and sex-matched WT controls, allowing us to control for task experience. Our longitudinal approach may have also had carry-over effects that impacted later cognitive testing, which should be considered when evaluating group differences (or lack thereof) on these measures.

In conclusion, our findings indicate that the rat TgF344-AD model exhibits sex-dependent motivational and hedonic deficits that manifest early in adulthood, which may relate to the prodromal apathy phenotype previously identified as a clinical risk factor for accelerated cognitive decline in AD. Future research is needed to establish underlying neural mechanisms and test interventions to reduce apathy and improve wellbeing in AD.

## Data Availability

The raw data supporting the conclusions of this article will be made available by the authors, without undue reservation.
